# Supplementary material for: Histamine H1 receptors in dentate gyrus-projecting cholinergic neurons of the medial septum suppress contextual fear retrieval in mice
Source: Nat Commun. 2024 Jul 10;15:5805. doi: 10.1038/s41467-024-50042-4 (PMC11237085; doi:10.1038/s41467-024-50042-4)
Supplement: Supplementary file 1 — Supplementary Information [file 41467_2024_50042_MOESM1_ESM.pdf]

Supplementary Information

Histamine H_1 receptors in dentate gyrus-projecting cholinergic neurons of the medial septum suppress contextual fear retrieval in mice

Cheng et al.

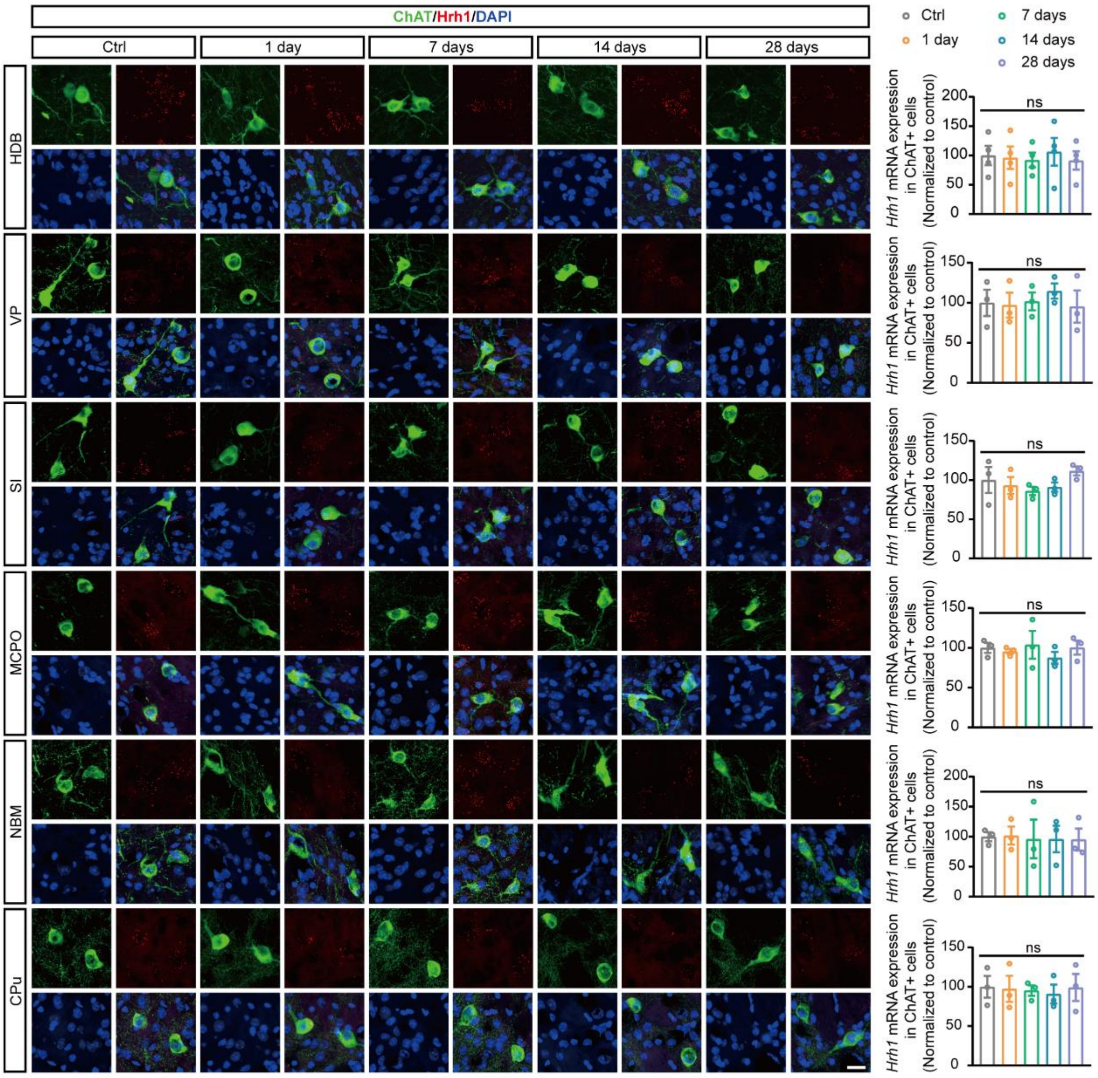

Supplemental figure 1. The *Hrh1* expression in the HDB/VP/SI/MCPO/NBM/CPu cholinergic neurons is unchanged after contextual fear conditioning. Left panels: representative images of RNAscope *in situ* hybridization of *Hrh1* mRNA together with immunostaining of choline acetyltransferase (ChAT) after contextual fear conditioning. Scale bar = 20 μm. Right panels: quantitative analysis of *Hrh1* mRNA expression in $ChAT^{+}$ cell. All data are presented as mean ± SEM. ns, nonsignificant. Further statistical information and source data are provided as a Source Data file.

HDB: nucleus of the horizontal limb of the diagonal band; VP: ventral pallidum; SI: substantia innominata; MCPO: magnocellular preoptic nucleus; NBM: nucleus basalis magnocellularis; CPu: caudate putamen (striatum).

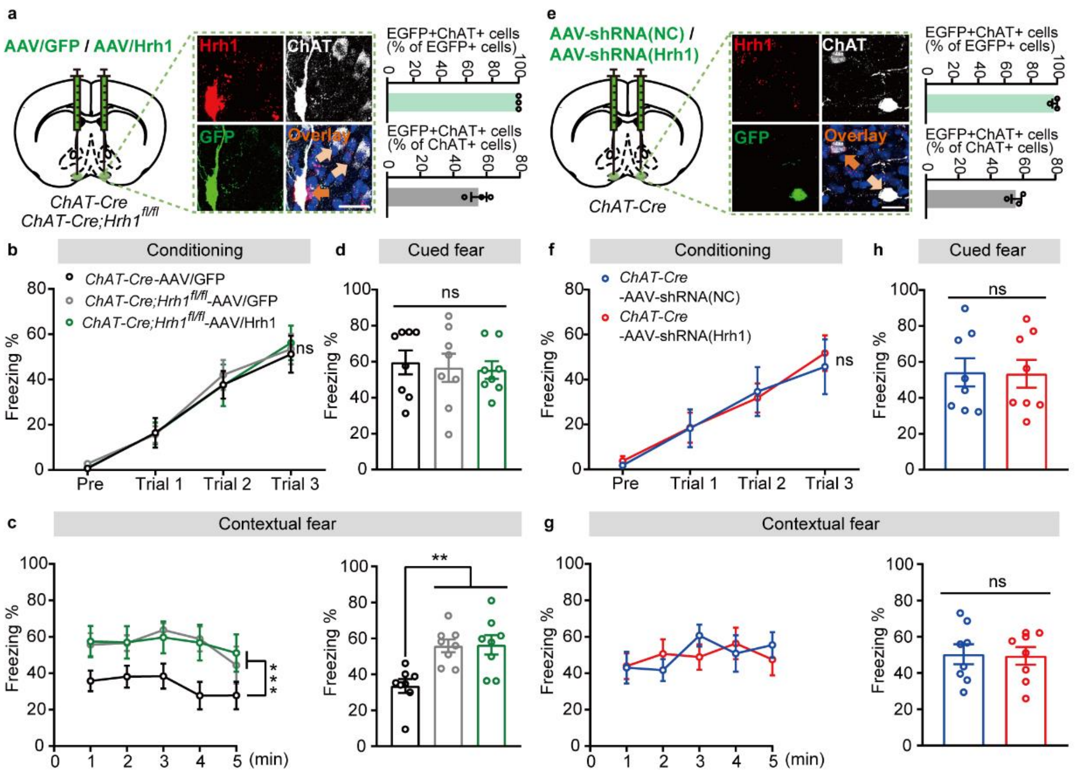

Supplemental figure 2. H_1R in HDB cholinergic neurons is not involved in contextual fear memory. a-d *ChAT-Cre;Hrh1$^{fl/fl}$* mice were injected with AAV-FLEX-Hrh1-GFP (AAV/Hrh1) or AAV-FLEX-GFP (AAV/GFP) in HDB. **a** Representative images of *Hrh1* (red), *GFP* (green) and ChAT (white) expression in the HDB of *ChAT-Cre;Hrh1$^{fl/fl}$* mice after the microinjection of AAV/Hrh1. The percentage of GFP+ cells co-expressing ChAT and percentage of ChAT+ cholinergic neurons co-expressing GFP in the HDB were quantified. Scale bar, 30 μm. n = 3 mice. **e-h** *ChAT-Cre* mice were injected with AAV-DIO-EGFP-shRNA(Hrh1) (AAV-shRNA(Hrh1)) or AAV-DIO-EGFP-shRNA(NC) (AAV-shRNA(NC)) in HDB. **e** Representative images of *Hrh1* (red), *GFP* (green) and ChAT (white) expression in the HDB of *ChAT-Cre* mice after the microinjection of (AAV-shRNA(Hrh1)). The percentage of GFP+ cells co-expressing ChAT and percentage of ChAT+ cholinergic neurons co-expressing GFP in the HDB were quantified. Scale bar, 30 μm. n = 3 mice. **b, f** The curve of freezing level during the exploration period and each trial on the conditioning day. **c, g** The curve of freezing level in each minute (left panel) and the percentage of freezing time during the contextual fear memory retrieval (right panel). **d, h** The percentage of freezing time during the cued fear memory retrieval. All data are presented as mean ± SEM. $^{**}P<0.01$,

ns, nonsignificant. Further statistical information and source data are provided as a Source Data file.

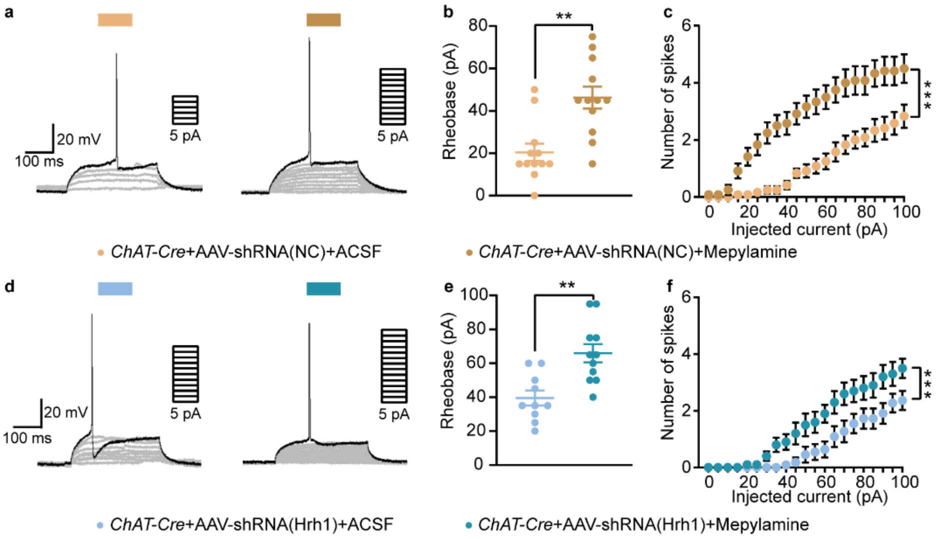

Supplemental figure 3. The excitability of MS cholinergic neurons bathed in either ACSF or H₁R antagonist at baseline or following H₁R knockdown. a-b, d-e Threshold current to elicit action potential with the increase of injected currents in MS cholinergic neurons bathed with ACSF or H₁R antagonist Mepyramine. **c, f** Spike numbers with the increase of injected currents in MS cholinergic neurons bathed with ACSF or H₁R antagonist Mepyramine. All data are presented as mean ± SEM. ***P*<0.01, ****P*<0.001, ns, nonsignificant. Further statistical information and source data are provided as a Source Data file.

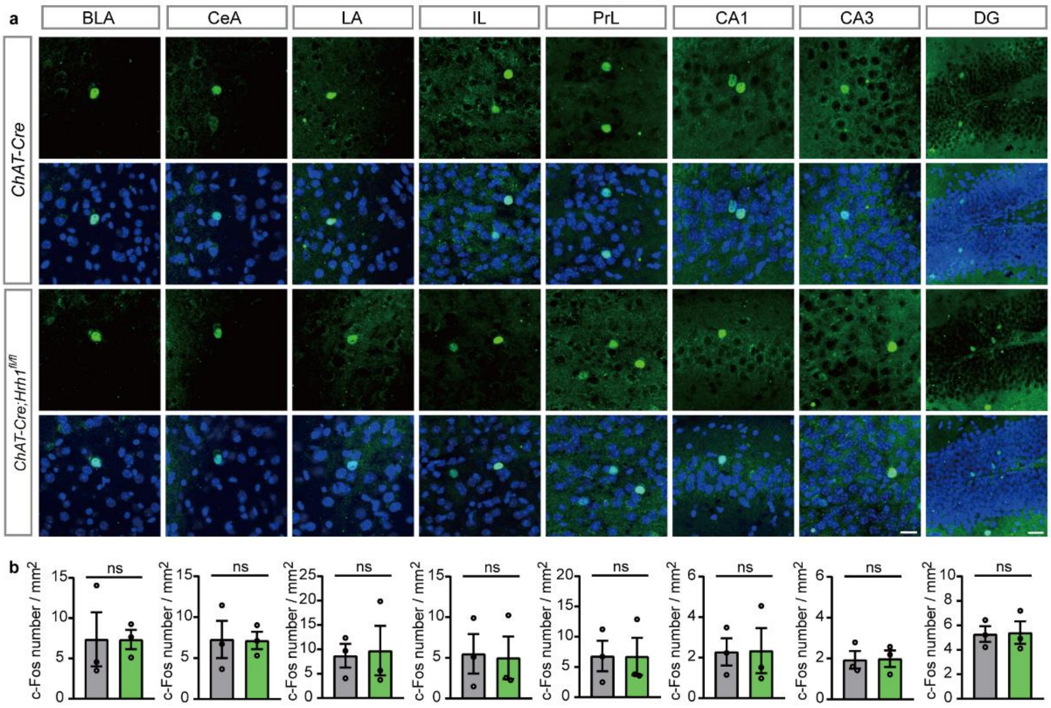

Supplemental figure 4. The c-Fos expression of *ChAT-Cre* and *ChAT-Cre;Hrh1$^{fl/fl}$* mice trained but not re-exposed. a Representative images of c-Fos expressing neurons in the amygdala, medial prefrontal cortex and hippocampus of *ChAT-Cre* and *ChAT-Cre;Hrh1$^{fl/fl}$* mice trained but not re-exposed. Left panel: scale bar = 20 μm. Right panel: scale bar = 30 μm. **b** The percentage of c-Fos expressing neurons of the indicated areas. All data are presented as mean ± SEM. ns, nonsignificant. Further statistical information and source data are provided as a Source Data file.

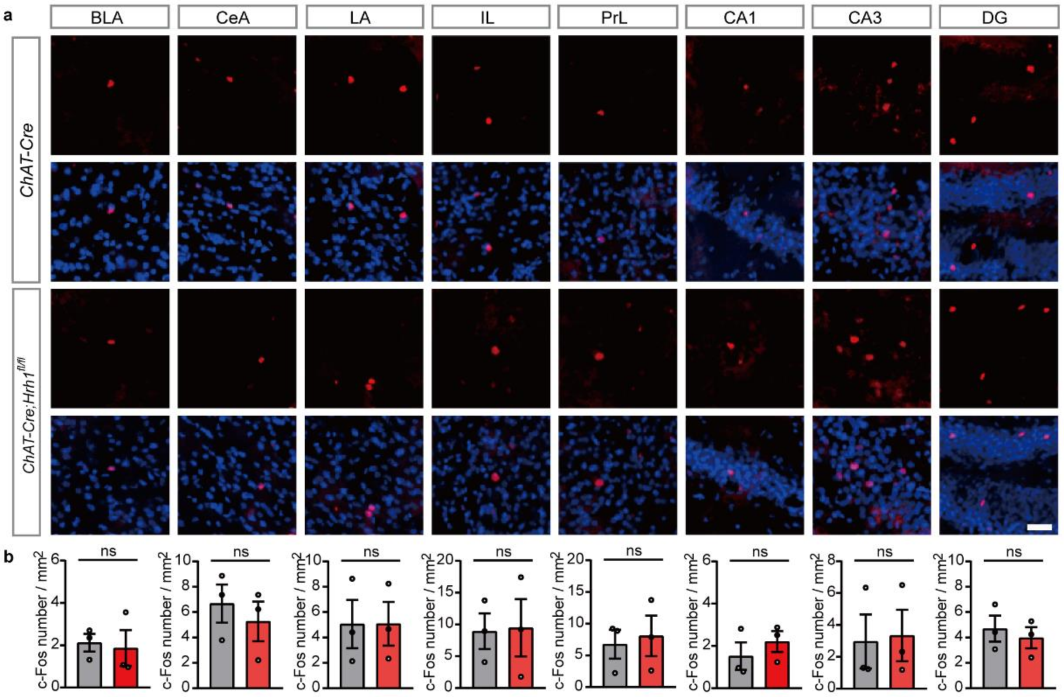

Supplemental figure 5. Deletion of H_1R in cholinergic neurons has no effect on c-Fos expression in the basal state. a Representative images of c-Fos expressing neurons in the amygdala, medial prefrontal cortex and hippocampus of *ChAT-Cre* and *ChAT-Cre;Hrh1$^{fl/fl}$* mice in the basal state. Scale bar = 30 μm. **b** The percentage of c-Fos expressing neurons of the indicated areas. All data are presented as mean ± SEM. ns, nonsignificant. Further statistical information and source data are provided as a Source Data file.

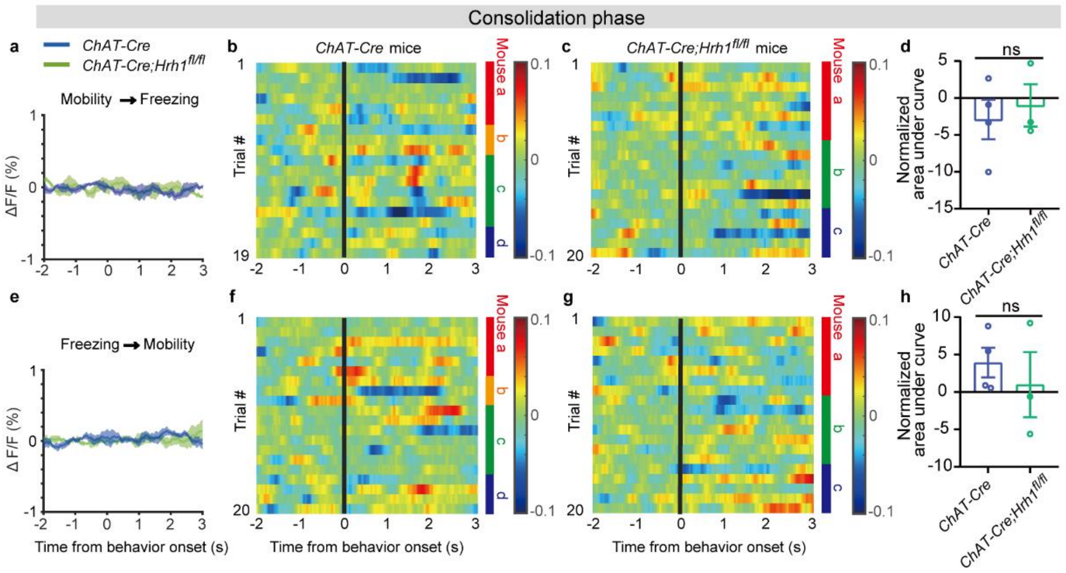

Supplemental figure 6. The *ChAT-Cre;Hrh1$^{fl/fl}$* mice exhibit normal functional acetylcholine release to DG neurons during consolidation of contextual fear memory. a Averaged plots of ACh release aligned to the onset of freezing bouts of both *ChAT-Cre* mice and *ChAT-Cre;Hrh1$^{fl/fl}$* mice. **b, c** Heatmap representations of ACh release aligned to the onset of freezing bouts of *ChAT-Cre* mice **(b)** and *ChAT-Cre;Hrh1$^{fl/fl}$* mice **(c)**. Color bars at the right of each heatmap represent different individual mice. **d** Normalized AUC of DG acetylcholine release for freezing bouts. **e** Averaged plots of ACh release aligned to the onset of mobility bouts of both *ChAT-Cre* mice and *ChAT-Cre;Hrh1$^{fl/fl}$* mice. **f, g** Heatmap representations of ACh release aligned to the onset of mobility bouts of *ChAT-Cre* mice **(f)** and *ChAT-Cre;Hrh1$^{fl/fl}$* mice **(g)**. Color bars at the right of each heatmap represent different individual mice. **h** Normalized AUC of DG ACh release for mobility bouts. All data are presented as mean ± SEM. ns, nonsignificant. Further statistical information and source data are provided as a Source Data file.

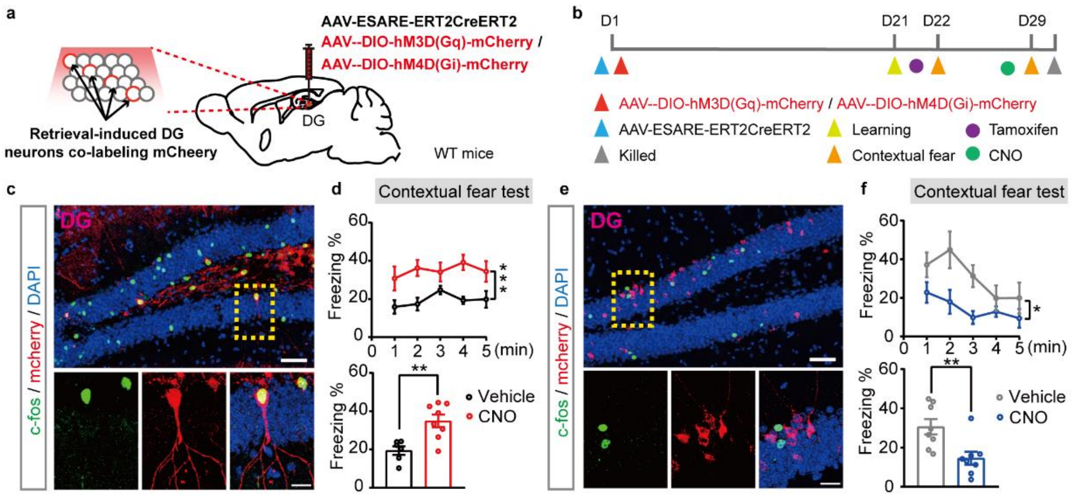

Supplemental figure 7. The retrieval-induced neurons in DG can bidirectionally regulate the retrieval of contextual fear memory. a Schematic for expression of hM3Dq or hM4Di in retrieval-induced neurons in DG for chemogenetic manipulation. **b** The timeline of the chemogenetic manipulation experiment. **c** Representative images from the CNO group show c-Fos and mCherry immunostaining in DG. Upper panel: scale bar = 50 μm. Lower panel: scale bar = 20 μm. **d** The curve of freezing level in each minute (upper panel) and the percentage of freezing time during the contextual fear memory retrieval (lower panel). **e** Representative images show the absence of colocalization between c-Fos and mCherry in DG after CNO administration. Upper panel: scale bar = 50 μm. Lower panel: scale bar = 20 μm. **f** The curve of freezing level in each minute (upper panel) and the percentage of freezing time during the contextual fear memory retrieval (lower panel). All data are presented as mean ± SEM. **P*<0.05, ***P*<0.01, ****P*<0.001, ns, nonsignificant. Further statistical information and source data are provided as a Source Data file.

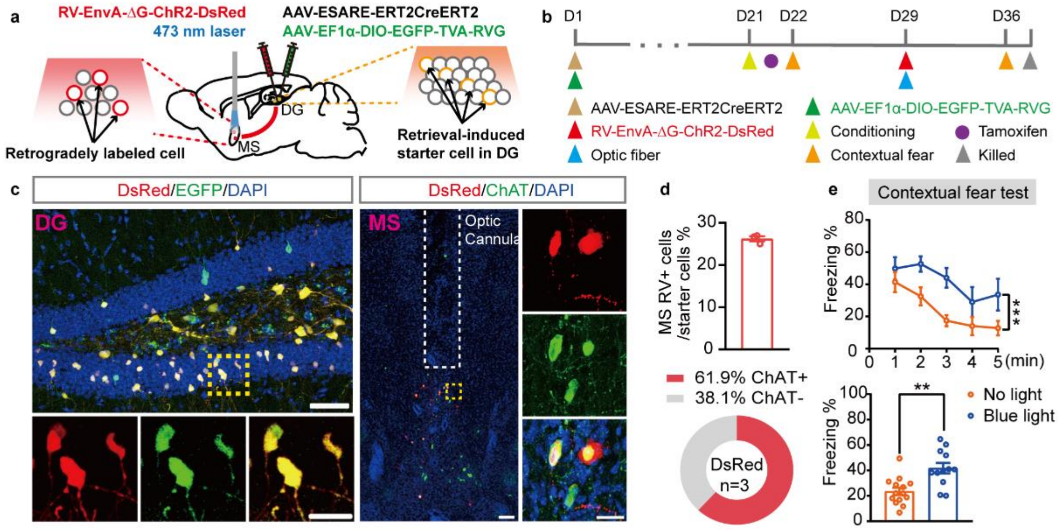

Supplemental figure 8. Optogenetic activation of MS inputs to the retrieval-induced neurons in DG elicits enhanced contextual fear memory. a Schematic for optogenetic activation of MS neurons that project to retrieval-induced neurons in DG. **b** The timeline of optogenetic activation experiment. **c** Left panel: Representative images of EGFP and DsRed doublelabeled starter cells in the DG. Upper panel: scale bar = 50 μm. Lower panel: scale bar = 20 μm. Right panel: Representative images show ChAT and DsRed (expressed by retrogradely labeled cells) expression in the MS. Left panel: scale bar = 100 μm. Right panel: scale bar = 20 μm. **d** Upper panel: Quantification of rabies-labeled MS neurons. Lower panel: Percentage of rabies-labeled, immunochemically identified MS cholinergic neurons. n=3 mice. **e** The curve of freezing level in each minute (upper panel) and the percentage of freezing time during the contextual fear memory retrieval (lower panel). All data are presented as mean ± SEM. $^{**}P<0.01$, $^{***}P<0.001$. Further statistical information and source data are provided as a Source Data file.

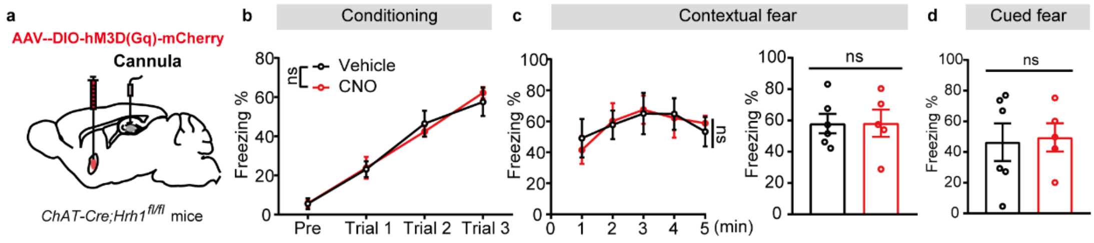

Supplemental figure 9. Chemogenetic activation of MS cholinergic neurons during the consolidation phase has no effect on contextual fear memory of *ChAT-Cre;Hrh1$^{fl/fl}$* mice. a Schematic diagram of microinjection of AAV-DIO-hM3D(Gq)-mCherry in the MS in *ChAT-Cre;Hrh1$^{fl/fl}$* mice. **b** The curve of freezing level during the exploration period and each trial on the conditioning day. **c** The curve of freezing level in each minute (left panel) and the percentage of freezing time during the contextual fear memory retrieval (right panel). **d** The percentage of freezing time during the cued fear memory retrieval. All data are presented as mean ± SEM. ns, nonsignificant. Further statistical information and source data are provided as a Source Data file.

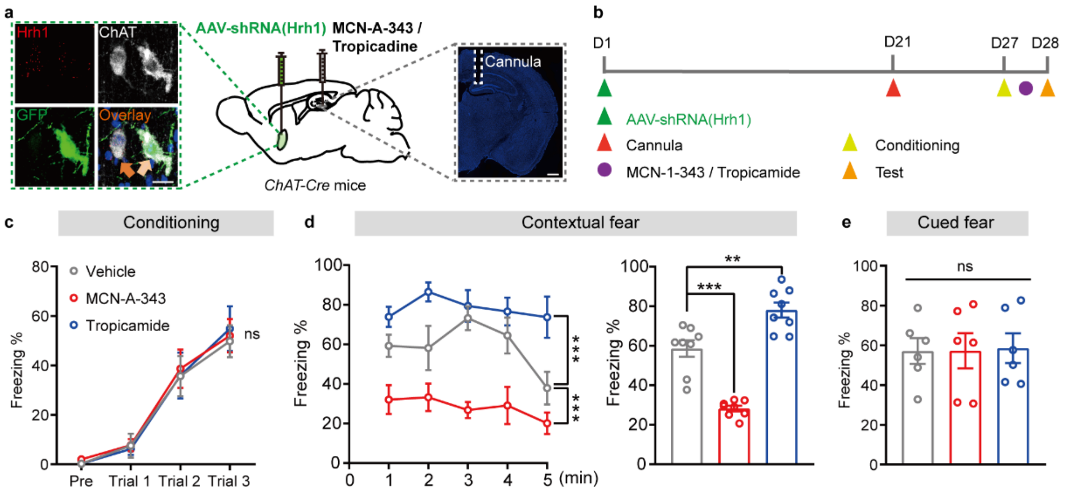

Supplemental figure 10. M_4R in the DG is involved in the enhanced contextual fear retrieval induced by H_1R deficiency in MS cholinergic neurons. a Left panel: representative images of the AAV-shRNA(Hrh1) expression in the MS. Scale bar = 20 µm. Right panel: representative image of cannula placement (indicated by the white dotted line) in the DG. Scale bar = 500 µm. **b** The timeline of experiment. **c** The curve of freezing level during the exploration period and each trial on the conditioning day. **d** The curve of freezing level in each minute (left panel) and the percentage of freezing time during the contextual fear memory retrieval (right panel). **e** The percentage of freezing time during the cued fear memory retrieval. All data are presented as mean ± SEM. $^{**}P<0.01$, $^{***}P<0.001$, ns, nonsignificant. Further statistical information and source data are provided as a Source Data file.
